# CDAO-Store: Ontology-driven Data Integration for Phylogenetic Analysis

**DOI:** 10.1186/1471-2105-12-98

**Published:** 2011-04-15

**Authors:** Brandon Chisham, Ben Wright, Trung Le, Tran Cao Son, Enrico Pontelli

**Affiliations:** 1 Department of Computer Science, New Mexico State University, Las Cruces, New Mexico, USA

## Abstract

**Background:**

The *Comparative Data Analysis Ontology (CDAO) *is an ontology developed, as part of the EvoInfo and EvoIO groups supported by the National Evolutionary Synthesis Center, to provide semantic descriptions of data and transformations commonly found in the domain of phylogenetic analysis. The core concepts of the ontology enable the description of phylogenetic trees and associated character data matrices.

**Results:**

Using CDAO as the semantic back-end, we developed a triple-store, named *CDAO*-*Store*. CDAO-Store is a RDF-based store of phylogenetic data, including a complete import of TreeBASE. CDAO-Store provides a programmatic interface, in the form of web services, and a web-based front-end, to perform both user-defined as well as domain-specific queries; domain-specific queries include search for nearest common ancestors, minimum spanning clades, filter multiple trees in the store by size, author, taxa, tree identifier, algorithm or method. In addition, CDAO-Store provides a visualization front-end, called *CDAO*-*Explorer*, which can be used to view both character data matrices and trees extracted from the CDAO-Store. CDAO-Store provides import capabilities, enabling the addition of new data to the triple-store; files in PHYLIP, MEGA, nexml, and NEXUS formats can be imported and their CDAO representations added to the triple-store.

**Conclusions:**

CDAO-Store is made up of a versatile and integrated set of tools to support phylogenetic analysis. To the best of our knowledge, CDAO-Store is the first semantically-aware repository of phylogenetic data with domain-specific querying capabilities. The portal to CDAO-Store is available at http://www.cs.nmsu.edu/~cdaostore.

## Background

The *CDAO-Store *is a novel portal aimed at facilitating the storage and retrieval of phylogenetic data. The novelty of CDAO-Store lies in the use of a *semantic-based *approach to the storage and querying of data, building on established ontologies for the semantic annotation of data. This approach enables scientists to overcome the restrictions imposed by the use of specific data formats-thus, facilitating inter-operation among phylogenetic analysis applications-and makes it possible to design and implement more meaningful domain-specific queries.

Phylogenetic trees have gained a central role in modern biology. Trees provide a systematic structure to organize evolutionary knowledge about diversity of life. Trees have become fundamental tools for building new knowledge, thanks to their explanatory and comparative-based predictive capabilities. Evolutionary relationships provide clues about processes underlying biodiversity and enable predictive inferences about future changes in biodiversity (e.g., in response to climate or anthropogenic changes). Phylogenies are used with increasing frequency in several fields, e.g., comparative genomics [1], meta-genomics [2], and community ecology [3].

Below we highlight some of the core technologies that have facilitated the use of phyloinformatics solutions in various areas of biology and some of the issues faced.

*Phylogenetic Repositories*: the development of new knowledge relies on the ability to share and reuse data and results. To meet this goal, several large repositories of phylogenetic data have been developed and deployed. These repositories accepts submissions of diverse types of phylogenetic data, including different types of trees (e.g., trees of genes, trees of species) along with the data (e.g., character data) used to generate them. Repositories like Tree-BASE [4] store phylogenetic data along with metadata describing publications and published analyses, and offer querying capabilities to retrieve data from different studies, for comparison, combination, and reuse. TreeBASE accepts submissions of phylogenies and associated data in NEXUS format

Another related project is the *Tree of Life Web *project [5], a collaborative web portal that provides a hierarchical organization, in the form of an evolutionary tree of life, to web pages providing information about characteristics and biodiversity of different groups of organisms.

*Data Inter-operation*: Data reuse however is not practically possible without data inter-operation. Data tied to a particular tool, or worse, a particular version of a given tool, provides limited value to users of a repository. Ideally, repositories should supply their clients with results in a maximally compatible format that does not limit the client to the use of a particular piece of software. This issue is of particular interest to the evolutionary biology community. Several competing formats, e.g., NEXUS [6], nexml[7], phyloxml[8], PHYLIP [9], exist for representing phylogenies and the underlying molecular and morphological character data. Additionally, there are no commonly accepted methods for applying annotations to branches in a phylogeny, or for describing evolutionary models. Also, other meta-data, such as provenance, are not commonly handled.

Observe that the use of automated techniques for addressing the data inter-operation problem requires the availability of the *semantics *of data, i.e., a formally specified description of the meaning of data. The semantics of data allows software tools to correctly map data items encoded in different formats.

*Semantics*, *Ontologies*, *Triple-Stores*: In order to gain full effectiveness, data inter-operability cannot be restricted to exchange of data, but it needs to rely on the exchange of *semantics*. While data formats capture the syntax of data (e.g., for data exchange), explicit semantics is necessary (e.g., [10]) for interpretation, repurposing and application of phylogenetic data. The presence of an explicit representation of the semantics of data enables the develoment of of provably correct tools to perform data exchange between different data formats, and the integration of data arising from diverse sources. In recent years, semantic descriptions in the biomedical domains have predominantly built on the use of domain-specific *ontologies *[11,12]-enabling the formalization of domain knowledge in terms of domain *concepts *and *relations *among concepts.

A domain ontology for the field of phylogenetic analysis, called the *Comparative Data Analysis On-tology (CDAO)*, has been recently introduced [10].

A number of technologies and standards have been introduced to enable the representation and use of ontologies. The *Web Ontology Language (OWL) *[13] is a formal language that has been developed for publishing and sharing ontologies on the web. OWL enables the description of an ontology in terms of a collection of classes of entities (commonly referred to as *concepts*), organized in a taxonomy, and a collection of relations among entities (commonly referred to as *properties*). Properties are binary relations among two concepts. For example, the CDAO ontology contains, among the others, two concepts used in the description of phylogenies, called Edge-representing one edge of a phylogeny-and EdgeLength-representing the length of an edge; these two concepts are related by a property called has_Annotation, which associates an element of EdgeLength to each element of Edge.

The instances of an ontology-i.e., the concrete objects belonging to the classes described in the ontology and the specific connections created by the properties-are typically described in the form of *triples*-depicting two entities being linked by a property. For example, the triple (node_Arabidopsis_thaliana_AAD31, instance_of, Node) describes the fact that the entity named node_Arabidopsis_thaliana_AAD31 belongs to the class Node; similarly, the triple (node_Arabidopsis_thaliana_AAD31, belongs_to_Edge_as_Child, edge_AAD31_15) describes the fact that the entity node node_Arabidopsis_thaliana_AAD31 is associated to to the edge named edge_AAD31_15 in the phylogeny, and it is in the descendant position of the edge.

The World Wide Web consortium has formalized an XML format for the description of triples, called the *Resource Description Framework (RDF) *[14]; being an XML format, RDF provides an unambiguous format for storing and exchanging triples. We refer to a repository of ontology instances, expressed as triples, as a *triple-store*.

*Domain-specific Querying*: Domain-specific querying is also an important feature of a phylogenetic repository (see, e.g., [15])-i.e., the ability of the repository to provide direct access to queries that are specific to a given application domain, without requiring the user to encode them using a domain-independent query language (e.g., SQL). This level of query support helps investigators to easily pose questions to the repository that might be otherwise difficult or impossible to express in a general purpose query language. Several approaches have been proposed to support domain-specific querying in the domain of phylogenetic analysis. TreeBASE provides six predefined types of searches of their repository- i.e., search by taxon, by author, by citation, by study accession number, by matrix accession number, and by structure. These searches are mostly based on the syntactic content of the data, and not dissimilar from traditional relational database queries.

The study described in [15] identifies six main areas of studies that involve the use of phylogenetic data-*general/casual uses*, *visualization studies, database studies, super-tree algorithmic studies, simulation and contests studies, and comparative genomic studies*. A set of standard query types necessary to support the needs of these six classes of investigations have been identified and discussed by the authors of [15].

### CDAO

The *Comparative Data Analysis Ontology (CDAO) *http://www.evolutionaryontology.org[10] provides a formal ontology for describing phylogenies and their associated character state matrices. CDAO has been developed as part of the *Evolutionary Informatics (EvoInfo) *https://www.nescent.org/wg_evoinfo/Main_Page working group, sponsored by the National Evolutionary Synthesis Center.

**Figure 1:**
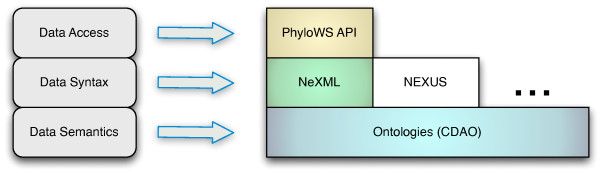
**The EvoIO Stack**. This is the structure of the EvoIO stack developed by he EvoInfo working group of the National Evolutionary Synthesis Center.

CDAO provides the semantic component of a data representation and inter-operation stack for phyloinformatics, known as the *EvoIO stack *[16]-along with a data exchange format, called nexml[7], and a phyloinformatics web services API, known as PhyloWS [17]. CDAO forms the base of this stack, defining the semantics for the data represented in nexml files, or otherwise supplied by services implementing this set of standards. Figure 1 illustrates the EvoIO stack.

**Figure 2:**
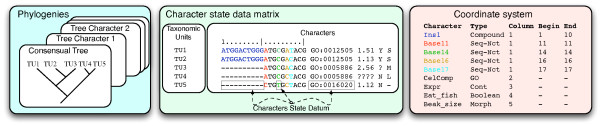
**The Principle View of OTUs and Characters**. This figure summarizes the core concepts from phylogenetic analysis that are captured by the CDAO ontology.

**Figure 3:**
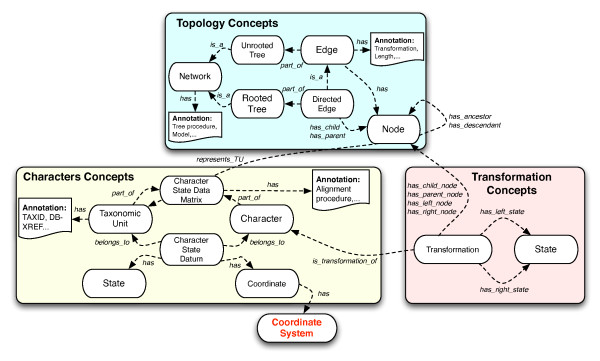
**Snapshot of the Key concepts of CDAO**. This figure provides a very small summary of the core concepts and relations described in CDAO.

CDAO is implemented as a formal ontology encoded in OWL. It provides a general framework for talking about the relationships between taxa, characters, states, their matrices, and associated phylogenies. The ontology is organized around five central concepts (see also Figure 2): *OTUs, characters, character states, phylogenetic trees, and transitions*. The key concepts and their mutual relationships within CDAO are illustrated in Figure 3. A phylogenetic analysis starts with the identification of a collection of *operational taxonomic units (OTUs)*, representing the entities being described (e.g., species, genes). Each OTU is described, in the analysis, by a collection of properties, typically referred to as *characters*. In phylogenetic analysis, it is common to collect the characters and associated character states in a matrix, the character state matrix, where the rows correspond to the different OTUs and the columns correspond to the characters.

In evolutionary biology, phylogenetic trees and networks are used to represent paths of descent-with-modification, capturing the evolutionary process underlying the considered OTUs. Since evolution moves forward in time, the branches of a tree are typically directed. The terminal nodes are anchored in the present, as they represent observations or measurements made on existing organisms. The internal nodes represent common ancestors, with the deepest node as the root node of the tree. The restriction that each node has at most one immediate ancestor reflects the assumption that evolutionary lineages, once separated, do not fuse (e.g., because of the assumption of the *biological species concept *based on reproductive isolation). Branching is considered to be a binary process of splitting by speciation (or gene duplication, in the case of molecular sequences). Even with terminal nodes anchored in the present, it may be impossible to infer the direction of each internal branch, in which case the tree may be referred to as an unrooted tree or as a network. Even the restriction of single parentage may be occasionally abandoned (e.g., in the case of lateral transfer or reticulate evolution).

As a general framework, CDAO supplies general classes and relations between those classes that can be further specialized to meet the needs of a specific application-*Beak length *might be introduced as a new concept that specializes CDAO's *Standard *character type.

nexml

nexml[7] is a file format for exchanging data containing character state data matrices and phylogenies. Its syntax is defined in terms of an XML schema (i.e., a grammar describing the legal structure of an XML document) and the semantics of its elements are defined in terms of CDAO classes-thus allowing an easy mapping of data files to CDAO instances. This property is also important to enable the effective use of nexml as a data exchange medium, since its semantics can be agreed upon by both the provider and recipient of a dataset.

A typical nexml specification [18] is embedded within the element <nexml>, and contains the description of phylogenies and character data matrices. The elements <otus> are analogous to the TAXA block in NEXUS, and they are used to describe the identifiers and (optionally) labels of all the relevant taxonomic units employed in the investigation. The elements <characters> play a role analogous to the CHARACTERS block in NEXUS, allowing the description of the character state matrices. nexml allows the use of different formats, such as molecular sequences, categorical data, or continuous data. A difference from NEXUS is that more information per character can be specified; depefinding on the format, the matrices can be formed either by <matrix><row> elements or by <states><state> elements. The <tree> element is used to describe a phylogenetic tree, in a manner similar to GraphML [19]-i.e., by describing each tree as a sequence of <node> and <edge> elements. The <node> elements are used to describe the individual nodes of the tree, while the <edge> elements provide an explicit description of the connections among nodes. The edges of the tree are directed-i.e., each edge has a start point and an end point. A final element that deserves mention is <dict>: this element allows one to set up general attribute/value pairs, that can be attached to most elements of a nexml document, allowing the introduction of arbitrary meta-data for different elements of the data file.

An alternative XML format for the encoding of phylogenetic datasets is phyloxml[8]; this format allows the description of phylogenies (using the element phylogeny) described through the recursive use of the element clade. The format supports the description of various specialized properties, such as evolutionary events (e.g., duplication), and taxonomic information. Although CDAO-Store supports predominantly nexml, it includes a phyloxml converter, which allows exporting CDAO-Store data into phyloxml files, enabling the use of some of the tools already developed to process phyloxml data (e.g., the sophisticated PhyloBox visualization tool [20]).

### PhlyoWS

*PhyloWS (Phyloinformatics Web Services API) *is a standard for exposing phylogenetic data as web services. Web services are tools that can perform certain tasks, and whose execution can be programmatically requested using a standard Internet exchange protocol (i.e., HTTP) [21]. PhlyoWS specifically uses RESTful style web services, and implements a few well-known operations to relay data [22,23]. This works in a similar way as GET or POST for HTTP [23]. All PhlyoWS URIs begin with/phylows/as the standard delimiter. Then, based on the phylogenetic information being queried, a data structure will be given, such as taxon, tree, or study. This is followed by any specific identifiers needed for the query. For example, http://purl.org/phylo/treebase/phylows/tree/TB2:Tr3099?format=rdf is a way to access in-formation from TreeBASE using PhyloWS: when this URL is accessed, it returns the tree with the TreeBASE ID equal to 'Tr3099' in RDF format [24]. A specification for PhyloWS can be found in [22].

## CDAO-Store Implementation

CDAO-store builds on the EvoIO technology stack to provide a semantic-based repository of phylogenetic data, accessible through semantic web services and a domain-specific query language. As such, CDAO-Store primarily builds on the use of CDAO for the internal semantic-based representation of data and for the purpose of data querying, on the primary use of nexml for data exchange, and on the use of PhyloWS for the programmatic use of the store. Nevertheless, as highlighted next, CDAO-Store goes well beyond the EvoIO stack, supporting other data formats and querying mechanisms. The CDAO-store platform is open-source and is available as a SourceForge project, at http://sourceforge.net/projects/cdaotools.

**Figure 4:**
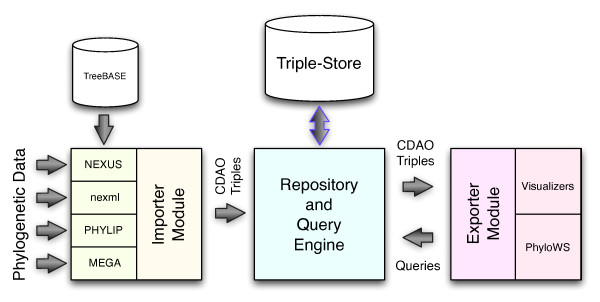
**Structure of CDAO-store**. This figure shows the overall structure of the implementation of the CDAO-store.

The implementation of CDAO-store is organized in three interconnected modules, as illustrated in Figure 4: a *data importer module*, a *repository module*, and an *exporter module*.

### Data Importer Module

The purpose of the *data importer *module is to import phylogenies and their associated data into the repository, automatically extracting their representations in terms of instances of the CDAO ontology. The *data importer *module can process phylogenetic data encoded in several commonly used data formats. The current implementation provides sub-modules that can extract CDAO instances from files encoded in NEXUS [6], nexml[7], PHYLIP [9], and MEGA [25].

The various parsing sub-modules have been developed either from scratch, using combinations of C++ and XSLT style sheets, or using prebuilt libraries, such as the NEXUS Class Library (NCL, http://sourceforge.net/projects/ncl). The data importer module is also designed to enable the processing of the content of the TreeBASE http://www.treebase.org repository-a popular repository of user-submitted phylogenies and associated generating data-importing the corresponding CDAO instances into the CDAO-store.

After reading each input file, the data importer module maps data from these files to an object model that mirrors CDAO classes, producing RDF/XML triples that can be deposited in the CDAO-store repository (i.e., passed to the repository module). The data importer module is also capable of mapping the object model back into any of the acceptable input data formats; this enables the use of the CDAO-store system for conversion among data formats.

### Repository Module

The repository module provides two core functionalities: *storage *and *querying*.

#### Storage

The repository module maintains a triplestore, used to store all the CDAO instances created, either through submitted user files or through processing of TreeBASE content. The triple-store is implemented in Python and uses the RDFlib http://www.rdflib.net module to store the RDF serializations of CDAO instances in a relational database (implemented using a MySQL database). The repository modules supports the execution of queries against the triple-store.

RDFlib provides an excellent balance between flexibility, simplicity, and power for CDAO-Store. It is flexible, allowing nearly seamless loading of RDF data from a variety of sources through a uniform interface. It also has a very flexible query interface, providing not only a SPARQL query processor, but also the ability to perform more complex operations, e.g., iteration over a graph. The SPARQL interface is also particularly attractive, as it offers very flexible output formatting features, allowing us to customize the output format of each query by simply changing a format string, rather than having to write more extensive output processors. This is particularly important, considering our intention of offering different output formats (e.g., RDF, nexml).

Python is also an attractive choice as host language, because of its expressiveness. Python allows for a very terse implementation-for example, the entire query processing script is far less than 100 lines. This eased the implementation, helping us in moving quickly from concept to a working model, and allowing us to focus our efforts on the particular questions we sought to answer and the features we sought to support. This choice also kept us from getting locked into a particular language or platform for the entire project. In our system, only the query processing is written in Python. The user-visible web components are largely PHP, and a good deal of the PhyloWS interface is coordinated by shell or Perl scripts.

With regard to simplicity, RDFlib is a nearly zero-configuration system, requiring only the type of database to connect to and some connection information. It automatically manages the creation of tables, indices, and other entities, making it possible to treat the entire store as a persistent graph with almost no custom code. In spite of its simplicity, it also proved to be robust, allowing us to import the entire TreeBASE, encoded in the form of CDAO triples, without any difficulty and with good performance.

In addition to RDFlib, we considered alternative platforms for the implementation-in particular, we explored the use of OWL-API [26], Jena [27], and AllegroGraph [28]. The use of OWL-API and Jena would have required a great deal more effort to manage the configurations and a more extensive implementation to support our queries. The load startup/load time for the system would have made writing a custom server a necessity in order to handle requests in a timely fashion. An experimental comparison with OWL-API and Jena represents future work, that we intend to perform as soon as we have accumulated sufficient user queries from users of the CDAO-Store.

AllegroGraph provides a variety of attractive features, including built-in support for the Prolog programming language, but in terms of configuration and licensing was a less attractive option. Additionally, while it offers excellent performance, it demonstrated a number of bugs and configuration problems during some preliminary experiments conducted at the TDWG 09 meeting.

In our first implementation, we did not consider the use of RDF processors that employ denormalized schemas (e.g., RAP [29] and Jena); while denormalized schemas provide efficiency, they also appear to increase space consumption, which was one of our concerns-as CDAO may lead to very refined and detailed representations (especially for character state matrices) and to a large number of instances. RDFlib uses a normalized schema, maintaining tables and views for all objects, associative-box, identifiers, literal-properties, literals, namespace binding s, relations, "relation or associative box," and "URI or literal object." This break-down helps in reducing redundant information and space, and facilitates the use of hashing to reduce column sizes. Nevertheless, the literature has recently reported significant performance differences in favor of denormalized approaches (e.g., Jena2), thus suggesting the need to explore in the future one of these alternative platforms [30]; observe that a platform like RAP can be introduced in CDAO-Store with ease.

#### Querying

The querying capabilities of the repository module can be accessed in two ways-through a web portal and through a programmatic interface. The portal is accessible through a standard web browser and provides fillable fields. The programmatic interface is accessible through the previously mentioned support for the PhyloWS web service interface. Both interfaces currently support the set of queries discussed next.

This set of queries is primarily drawn from the description given by Nakhleh et al. [15], that provides a characterization of a relevant set of domain-specific queries that are desirable for any repository of phylogenetic structures. The repository module supports all the types of queries identified in [15] (with only two exceptions, as mentioned later). This is a diverse set of queries, ranging from queries that require a simple database search, to queries that involve complex reasoning over tree structures. The domain-specific types of queries are:

1. Determine all the phylogenies containing a given set of taxa-e.g., locate all trees containing the taxonomic units named Ilex anomala and Ilex glabra;

2. Determine the relationships among a set of taxa in all phylogenies (query not supported);

3. Determine the minimum spanning tree/clade for a given set of taxa-e.g., locate the minimum spanning clade in the tree Tree3099 for the taxonomic units Ilex anomala and Ilex glabra;

4. Determine all phylogenies constructed using a given inference method-e.g., locate all the phylogenies constructed using the program PAUP*;

5. Determine all the phylogenies containing a set number of taxa-e.g., locate all the phylogenies with at most 25 clades;

6. Determine all the phylogenies produced by a given tool or author-e.g., locate all the phylogenies published by W. Piel;

7. Determine all phylogenies satisfying a given property-e.g., locate all the phylogenies that have diameter equal to 5;

8. Given a phylogeny *P *, a measure *m*, and a quantity *q*, determine all the phylogenies that are at distance *q *from *P *according to the measure *m *(e.g., for the purpose of clustering phylogenies that are "close" to a given tree);

9. Given a model of evolution, determine all the phylogenies that have been constructed using such model of evolution-e.g., identify all the phylogenies that have been constructed using Jukes-Cantor model for estimating distance;

10. Given a measure, return statistics about the measure in the phylogenies present in the repository-e.g., determine the distribution of tree lengths;

11. Given a type of data and a set of taxa, determine all the phylogenies on the set of taxa that have been constructed using the specified type of data-e.g., determine all phylogenies built using DNA sequences.

To address these different types of queries, the query system is divided into two primary modules:

• The RDFlib has been linked to a SPARQL [31] engine and an OWL reasoner, Pellet http://pellet.owldl.com/, enabling the execution of standard SPARQL queries to access the data in the triple-store. This allows the implementation of queries that require simply searching the content of the repository for triples containing a particular data item.

• Other types of queries are beyond the expressive power of the standard SPARQL query language-due to SPARQL's inability to query hierarchical structures of unknown depth, to query transitive relations (such as those used to describe paths in a phylogeny), the lack of support for some aggregate functions (e.g., SUM, needed to implement statistical queries like queries of type 10), and the relatively limited support provided by reasoners like Pellet in handling certain features of OWL ontologies (e.g., Pellet provides an inconsistent behavior in handling property chaining, which is used in CDAO to define the descendant relation within a phylogeny).

In order to support queries requiring these features, the repository module has the capability of mapping CDAO tree and network structures, stored in the triple-store, to corresponding representations of trees and networks in *Prolog *[32], a popular rule-based programming language for knowledge representation and reasoning. The choice of Prolog is suggested by its natural ability to represent and manipulate tree and graph structures, encoded as logical terms, and the ability to elegantly and efficiently address tasks involving transitive closures and aggregations. Thus, the remaining types of queries are implemented using Prolog rules.

Table 1 maps each type of query to the corresponding implementation method-the interested reader is also referred to [33] for details of how the various types of queries are mapped to Prolog and SPARQL. For the queries that are not supported, the primary cause is the lack of a precise specification of the query, or the lack of relevant data in the repository. For example, in the discussion of queries of type 2, the set of relationships one might be interested in having returned was not fully specified in the original article [15]. Finally, the availability of a SPARQL interface enables the user to submit also arbitrary user-defined queries, as long as these are expressible as SPARQL queries-through the PhyloWS web service interface.

**Table 1:** Implementation Methods for Queries

Query #	Implementation Method
#1	SPARQL

#2	Not Supported

#3	Prolog

#4	SPARQL

#5	Prolog

#6	SPARQL

#7	Prolog

#8	Prolog

#9	Not Supported

#10	SPARQL

#11	Prolog

The table illustrates the implementation method employed for each one of the various classes of queries discussed in [15].

### Exporter Module

The goal of the exporter module is to provide interactions with the user. The module provides three main interaction mechanisms: a *web portal*, a *web service interface*, and a set of *visualization tools*.

The web portal offers an HTML interface to interact with the repository. The interface allows the on-line submission of queries, the ability to browse the content of the triple-store, and forms to submit new data sets to the triple-store. The web portal allows also one to make annotations about a dataset, a general project space, a set of data sets of interest. These annotations can be from CDAO, Dublin-Core, or from a user-supplied source of annotation types (i.e., another ontology).

The web service interface is an implementation of the PhyloWS protocol; this is realized by a collection of scripts, capable of generating the necessary SPARQL/Prolog queries to be submitted to the repository module.

The visual interface, called CDAO-Explorer, provides two graphical visualization tools; one tool is used to provide a graphical representation of phylogenetic trees and networks, while the second one pro-vides graphical representations of character data matrices. The tools have been implemented using Java and the Prefuse visualization toolkit prefuse.org. Work is in progress to link existing visualization tools (e.g., PhyloBox and Nexplorer 3) to CDAO-Store.

The results of the queries can be retrieved as files in one of several data formats; currently, the repository allows retrieval of data in RDF/XML format (i.e., CDAO triples), nexml, NEXUS, phyloxml, Newick (for the representation of trees), GraphML (for the representation of trees), and Prolog.

## Results

### Web-Tools

The web tools provide a variety of querying and data access features for both human and programmatic access to data. It allows one to retrieve data sets by author name, tree identifier, taxon, algorithm, or method. It also supports computing the minimum spanning clade or the nearest common ancestor of a set of taxa. It also allows one to list trees conforming to certain measures. For example, finding all trees larger or smaller than a given size.

Our PhyloWS implementation is the basis for all the data access features of CDAO-Store. The other web components, and the CDAO-Explorer tool use it to access data. URI's are divided into three conceptual parts. The address of the store site, and path prefix http://www.cs.nmsu.edu/~cdaostore/cgi-bin/phylows, a query type (e.g., tree, matrix, msc, nca, size), and a parameters list. The specific parameters depend on the query type. For example, the msc ("Minimum Spanning Clade") and the nca ("Nearest Common Ancestor") query types expect a list of taxon id's separated by '/'. The listing query takes optional limit and offset parameters to paginate results. The size query takes a direction (greater, less, or equal), a criteria (node, internal, or leaf) and a size (a numeral).

### Performance

CDAO-Store, even though in its first release, has already reached a stable and reliable state. The store currently contains 93, 000, 153 triples, contributed by a porting of TreeBASE and by additional user submissions. The performance of the CDAO-Store on the various queries is dependent on the specific type and parameters of the query, but we have rarely encountered instances that would lead to response times higher than 120 seconds-and, for most of the queries provided to us by our alpha-testers, we observed average response times between 3 and 60 seconds. Table 2 reports some performance results for some of such queries.

**Table 2:** Execution Times for Some Sample Queries

Query Type	Description	Time
Type 3	Minimum Spanning Clade in Tree3099 for the OTUs Ilex anomala and Ilex glabra	1.82

Type 4	Trees Built using Parsimony Algorithms	6.12

Type 1	Trees Containing OTUs Ilex anomala and Ilex glabra	2.44

Type 6	Trees Authored by William Piel	5.42

Type 4	Trees Constructed using PAUP*	6.19

Type 5	Trees with at most 25 Nodes	32.58

Type 3	Basal Node of a Minimum Spanning Clade of Ilex anomala and Ilex glabra in Tree3099	0.91

Type 7	Trees having Width Equal to 13	15.91

The table illustrates the times, in seconds, for a collection of sample queries provided to the developers by a group of beta-testers.

### CDAO-Explorer

CDAO-Explorer has achieved a basic level of functionality. It provides search and visualization for both trees and matrices and a set of additional features not currently available in other related tools.

Annotation is an important part of CDAO-Explorer. It allows users to attach arbitrary an-notations to data items, as well as collections of resources. These annotations are expressed as instances of concepts drawn from CDAO or from any user-specified ontology. CDAO-Explorer also allows users to load or save custom data not in the repository and to export pictures of visualizations of trees and matrices.

The CDAO-Explorer platform is flexible, and in the long term it is expected to be an open platform for the integration of other visualization tools. For example, Nexplorer 3, which is capable of processing CDAO data, will be integrated with CDAO-Store. We have also demonstrated an integration of PhyloBox [20] as an alternative visualization interface, made possible by the ability to export data in phyloxml format.

#### Tree Viewer

**Figure 5:**
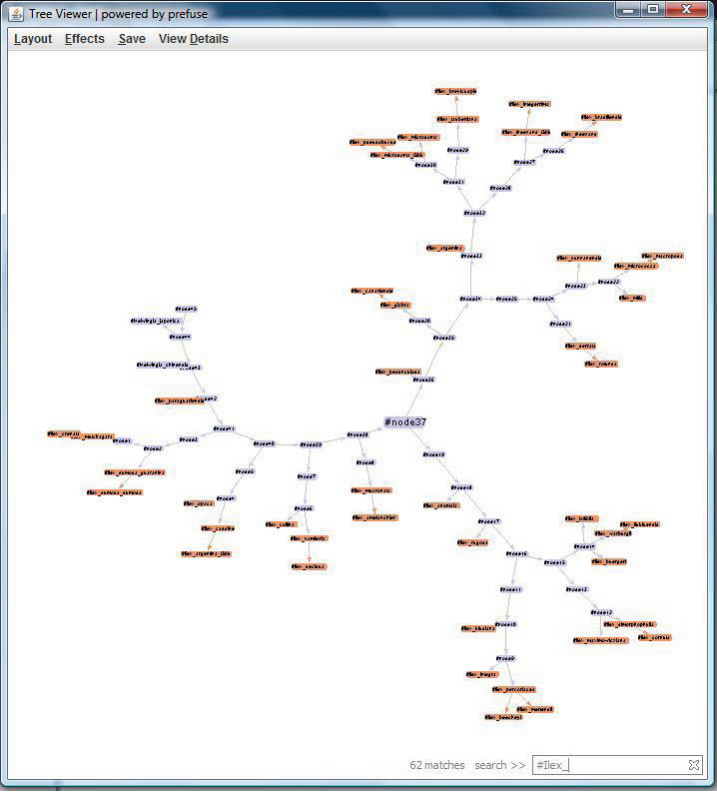
**Tree Viewer with search**. This is the TreeViewer Application displaying the tree Tree3099 from TreeBASE and searching for all nodes and edges with #Ilex_.

Tree Viewer is the graphical application used to display trees. It is built using the Prefuse visualization framework. Data from the CDAO triple-store (provided by the repository module) is converted into the GraphML format [19] and then supplied to the visualization application. Figure 5 shows a snapshot of the tree visualization.

The Tree Viewer provides several interesting features. The tool provides two different layouts for the tree to be displayed. By default, the Tree Viewer uses a *force layout*, which allows the nodes of the tree to "bounce" around as if pulled by strings until an equilibrium is reached. The second layout is called *node layout*, which resembles a more standard parent/child structured tree, going from left to right.

Another feature provided by the Tree Viewer is the ability to search the tree using the node and edge label names, highlighting all that successful matches found. For instance, a tree may have many nodes that have as part of its name #Ilex_. When this search is performed, all nodes with the label containing that will be highlighted. Labels for nodes are generally the taxa name for the corresponding taxonomic unit or, if it is an unknown internal node, will have the convention of being named #nodeX where X is some number. Edge labels are similar in that they are the labels of the two nodes combined as 'source_destination'.

It is also possible to view more specific details on a specific node or edge. Currently, the only detailed information available is the label. Finally, the Tree Viewer provides the option to save the tree visualization as a jpeg or png file.

#### Matrix Viewer

**Figure 6:**
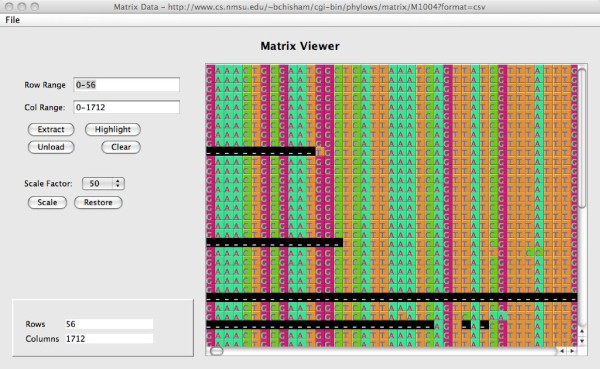
**Matrix Viewer**. This is a snapshot of the MatrixViewer component; it shows a DNA matrix, using colors to identify occurrences of identical nucleotides.

We have developed a custom framework for visualizing matrices. It assigns color codes to character states, allowing one to graphically appraise large matrices and quickly discover patterns in the source data. It allows users to scale matrices, select regions of a matrix to see in greater detail, and attach annotations to particular cells of a matrix. Figure 6 shows a snapshot of the Matrix Viewer.

### Related Work

#### TreeBASE

TreeBASE [4] is a relational database that stores phylogenies (of different nature), the associated alignments and character data used to derive the phylogenies, and several types of meta-data (e.g., authors and citations). The content of TreeBASE is community contributed and it is restricted to results of published studies. TreeBASE occupies a unique role, in providing access to both description of trees as well as corresponding generating data matrices. The spirit of TreeBASE is to enable retrieval of trees and data for study comparison, combination, and for reuse. The relational nature of the underlying repository enables a set of standard queries for accessing the repository. The original TreeBASE provided six forms of access to the repository (as discussed earlier in this paper); the newest release has expanded the submission formats (adding nexml as one of the supported formats, along with NEXUS), added support for the PhyloWS API, and connection to the PhyloWidget visualization tool.

#### Nexplorer

Nexplorer [34] is a tool developed to provide combined visualization of phylogenetic trees and associated character data matrices. The input to the Nexplorer is in NEXUS format; this could be either provided as a user-provided file or extracted via keyword search from a set of pre-processed data sets (e.g., 684 KOGs families and 7226 families from Pandit). The strength of Nexplorer is the ability to combine the visualization of phylogenies and of the associated data matrices. Nexplorer offers the ability to explore both leaves and internal nodes, and customize the visualization focusing on user-selected subsets of data. Differently from CDAO-Store, Nexplorer does not provide a direct connection to a repository and does not support data querying.

The latest release of Nexplorer (version 3) is currently under completion and it will be integrated with CDAO-Store, being capable of processing data encoded using CDAO.

#### PhyloWidget

PhyloWidget [35] is another application to visualize phylogenetic trees; the input is provided in Newick, NHX and NEXUS formats. The user interface enables a large level of interactivity and customization, including the ability to edit node labels and branch lengths, select and copy subtrees, and re-root the tree w.r.t. a selected node. The rendering engine can manipulate trees with thousands of nodes, producing effective representations (e.g., using rectangular, diagonal, and circular layouts).

## Discussion

The CDAO-store poses itself as the first semantically aware data repository for phylogenetic investigations. Its connection with TreeBASE and the ability to import arbitrary files from several different formats allows the repository to dynamically grow through community-contributed submissions; furthermore, the ability to provide additional annotations, driven by a formal ontology, allows community curation of data and facilitates the reuse of phylogenetic trees in different investigations. The CDAO-store provides the ability to integrate data from different experiments, through the use of a domain-specific query interface. In addition, the ability to convert to and from different data formats, using a intermediate semantic-based representation, guarantees semantically correct data format inter-conversion services.

Through these features, CDAO-store poses itself as a unique tool for data storage, reuse and inter-operation, overcoming limitations imposed by data formats and facilitating the development of workflows and (semi-)automated protocol implementations. The CDAO-store has been validated in preliminary community-driven experimentations (e.g., in the context of the first NESCent Phyloinformatics VoCamp, http://www.evoio.org/wiki/VoCamp1); several inter-operability demonstration projects are in progress, e.g., demonstrating inter-operation between TreeBASE and several visualization environments.

## Conclusions

### Current State

The CDAO-store tool set provides a robust foundation for a semantically aware, phylogeny resource. The query and translation services are well developed and based on an easily extensible framework to easily address additional development of features. The CDAO-Explorer portion of the store has achieved a good base-line functionality and provides a set of useful features to advance the current state of visualization of large data sets in this field. Also it provides a good proof-of-concept for integrating semantic information and other meta-data in a seam-less and natural way.

### Future Directions

Several features are currently being implemented to extend the capabilities and applicability of CDAO-store. For the web we plan to allow users to submit and execute their own *SPARQL *queries to our triple-store, enabling a wider range of queries than those supported by the current interface (currently this feature is supported only through the PhyloWS programmatic interface). CDAO-Explorer will include tighter integration between the tree and matrix visualizations, and also phase in support for describing processes and work flows, as part of its existing support for annotating sets of tree and matrix files.

We are also exploring mechanisms to provide a more direct integration between CDAO-store and TreeBASE, enabling regular updates of CDAO-store based on submissions to TreeBASE and enabling TreeBASE users to locate and access CDAO-store.

Finally, as the size of the repository increases, we intend to investigate whether RDFlib is an adequate triple-store system for the needs of CDAO-Store, or whether alternative platforms (e.g., RAP or Jena) would provide greater stability and performance.

## Availability and Requirements

**Project name**: CDAO-Store

**Project home page**: http://www.cs.nmsu.edu/~ cdaostore/

**Operating system(s)**: Linux, MacOS X, Unix

**Programming language**: C++, Java, Perl, PHP, Python, Prolog

**Other requirements**:

**License**: GPL

**Any restriction to use by non-academics**:

## Authors' contributions

BC focused on development of the web and database tools, and the integration of the tree and matrix and tree visualizers into the CDAO-Explorer application.

BW developed the tree viewer portion of the CDAO-Explorer tool, as well as updating the translator tool to accommodate the latest changes to the CDAO standard.

TL developed the MEGA format reader for the translator tool, as well as the matrix visualization tool.

TCS guided the development of the project.

EP led the development of the CDAO ontology and supervised the development of the project.

All authors read and approved the final version of this manuscript.
